# Size-Based Norfentanyl Detection with SWCNT@UiO-MOF Composites

**DOI:** 10.1021/acsami.3c17503

**Published:** 2023-12-26

**Authors:** Zidao Zeng, Meiirbek Islamov, Yiwen He, Brian A. Day, Nathaniel L. Rosi, Christopher E. Wilmer, Alexander Star

**Affiliations:** † Department of Chemistry, University of Pittsburgh, Pittsburgh, Pennsylvania 15260, United States; ‡ Department of Chemical & Petroleum Engineering, University of Pittsburgh, Pittsburgh, Pennsylvania 15260, United States; § Department of Electrical & Computer Engineering, University of Pittsburgh, Pittsburgh, Pennsylvania 15260, United States; ∥ Department of Bioengineering, University of Pittsburgh, Pittsburgh, Pennsylvania 15260, United States; ⊥ Clinical and Translational Science Institute, University of Pittsburgh, Pittsburgh, Pennsylvania 15260, United States

**Keywords:** field-effect transistor, opioid metabolite, carbon nanotube, UiO-66, UiO-67, MOF

## Abstract

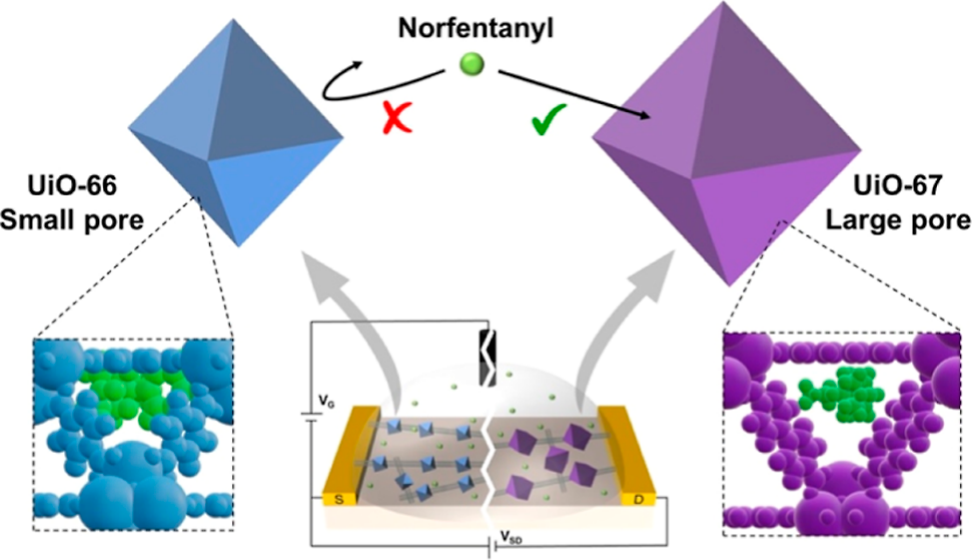

Single-walled carbon nanotube (SWCNT)@metal–organic framework (MOF) field-effect transistor (FET) sensors generate a signal through analytes restricting ion diffusion around the SWCNT surface. Four composites made up of SWCNTs and UiO-66, UiO-66-NH_2_, UiO-67, and UiO-67-CH_3_ were synthesized to explore the detection of norfentanyl (NF) using SWCNT@MOF FET sensors with different pore sizes. Liquid-gated FET devices of SWCNT@UiO-67 showed the highest sensing response toward NF, whereas SWCNT@UiO-66 and SWCNT@UiO-66-NH_2_ devices showed no sensitivity improvement compared to bare SWCNT. Comparing SWCNT@UiO-67 and SWCNT@UiO-67-CH_3_ indicated that the sensing response is modulated by not only the size-matching between NF and MOF channel but also NF diffusion within the MOF channel. Additionally, other drug metabolites, including norhydrocodone (NH), benzoylecgonine (BZ), and normorphine (NM) were tested with the SWCNT@UiO-67 sensor. The sensor was not responding toward NH and or BZ but a similar sensing result toward NM because NM has a similar size to NF. The SWCNT@MOF FET sensor can avoid interference from bigger molecules but sensor arrays with different pore sizes and chemistries are needed to improve the specificity.

## Introduction

Metal–organic frameworks (MOFs) are well-known for their nanoporous structure and high internal surface area, which led to their applications in gas storage and separation.^1−6^ Since most MOFs are not electrically conductive, intensive research has been conducted to develop conductive MOFs by introducing free charge carriers and creating low-energy transfer pathways in MOF structures.^7,8^ However, such strategies limit the variety of the MOF components and structure. Hybridizing MOFs with conductive carbon materials, such as single-walled carbon nanotubes (SWCNTs), multiwalled carbon nanotubes (MWCNTs), graphene, and reduced graphene oxide (rGO), offers a practical method to enable electrical applications for many traditional MOFs. This approach facilitated the evolvement of multiple nonconductive MOFs in electrical sensing, electrochemistry, and electrocatalysis.^9−14^ It is worth noticing that among the reported SWCNT/MOF hybrid material, most of the research centered around their application in electrochemistry.^15−19^ The utilization of SWCNT/MOF hybrid materials in electrical sensor applications was underexplored, despite the promising characteristics of SWCNTs.

SWCNTs offer a compelling combination of attributes essential for sensor development, including large surface-to-volume ratio, long-range conductivity, and inherent semiconductive properties.^20−22^ To leverage these advantages, SWCNT-based sensors typically incorporate selective chemical layers on the nanotube surfaces to enhance their specificity. These layers mostly include components such as metal nanoparticles, polymers, noncovalently attached small molecules, and covalent functionalizations.^23^ Utilizing MOFs as the selective layer is a novel and rational approach. The diverse functionalities of MOF ligands offer significant potential for chemical specificity, and the porosity of MOFs enables access to the surface of nanotubes, which is the most sensitive part of SWCNTs. Previous work from our group have shown improved chemiresistive gas sensing of ethanol, dimethyl methylphosphonate (DMMP), and H_2_ with SWCNT@ZIF-8,^24^ SWCNT@UiO-66-NH_2_,^25^ and SWCNT@Pd@HKUST-1^9^ composites, respectively. Furthermore, our recent report showcased the application of the SWCNT@MOF in liquid-gated FET devices for the first time. A series of carbohydrates with different molecular sizes was discriminated using FET devices made up of SWCNT@Cu_3_(HHTP)_2_.^26^

The sensing mechanism of the SWCNT@MOF FET devices is unique. Unlike conventional FET SWCNT sensors that rely on the electrostatic effect of the analyte to alter CNT conductance, SWCNT@MOF FET sensing operates by controlling gate capacitance through the inhibition of ion transportation to SWCNT surfaces, accomplished by the analyte molecules obstructing the MOF channels. This sensing mechanism was validated using a variety of carbohydrates with varying sizes that occupied the pores of Cu_3_(HHTP)_2_ MOF. As the pores of Cu_3_(HHTP)_2_ were most tightly packed by small-sized glucose molecules, largest current decrease was observed in the SWCNT@Cu_3_(HHTP)_2_ FET devices.^26^ In this work, we aim to explore the detection of a single analyte molecule using various SWCNT@MOF FET devices, each equipped with unique pore sizes. For this purpose, the UiO-6x MOF series is particularly suitable.

The UiO MOF series exhibits uniform structure, where a single zirconium oxide-cluster is ideally interconnected by 12 dicarboxylic ligands.^27^ By incorporation of benzene rings into the ligand, the UiO MOFs can be tailored to feature varied pore sizes while maintaining a consistent chemical environment within the channels. Additionally, the benzene ring enables numerous opportunities for functionalization, wherein functional groups can be introduced to slightly adjust both the pore size and chemical environment within the channels.^28,29^

Norfentanyl (NF) is the major metabolite from fentanyl, a potent and acute synthetic opioid.^30^ Detection of NF has earned huge research interests since illicit usage of fentanyl caused surge in overdose death in the United States.^31^ Research showed that NF had a longer detectable window after fentanyl administration and it presented in higher concentration compared to fentanyl in urine sample, which made NF a suitable marker to track fentanyl exposure.^32,33^ Detection of NF primarily relies on mass spectrometry-based techniques,^34−36^ which demands expensive instrumentation and specialized training. Traditional electrochemistry methods have not proven successful due to the redox-inactive nature of NF.^37^ Nevertheless, there have been a few successful demonstrations of electrical NF sensors achieved through the functionalization of NF biorecognition elements. Kumar et al. demonstrated the detection of three different opioid metabolites, including NF, using aptamer-functionalized graphene FET sensors (G-FET) with 2-digit pg/mL limit of detection.^38^ Shao et al. achieved detection at fg/mL level with a semiconducting-SWCNT-based FET biosensor (sc-SWCNT-FET) functionalized with NF antibody.^39^ However, it is important to note that these biorecognition elements require specific storage conditions, limiting their application in out-of-the-box and on-site scenarios. On the other hand, UiO-MOFs exhibit outstanding mechanical, thermal, and chemical stability.^27,40,41^ In tests for long-term stability, UiO-67 remained stable for up to 2 months when stored in water,^42^ while UiO-66 demonstrated remarkable prolonged stability, with no degradation observed over a period of 12 months.^43^ Reports also indicated that UiO-67 remained undegraded when kept dry,^44^ and UiO-66 exhibited no degradation even after exposure to high humidity for 28 days.^45^ This exceptional stability of UiO-MOFs is particularly advantageous for sensors intended for use in nonlaboratory environments. With pore size close to the NF molecule, UiO-MOFs are promising FET sensor materials for detecting NF. Herein, we studied the interaction between NF and SWCNT@UiO-MOF FET devices and report a novel size-based detection method for NF without functionalization with biorecognition elements.

## Results and Discussion

**Scheme 1:**
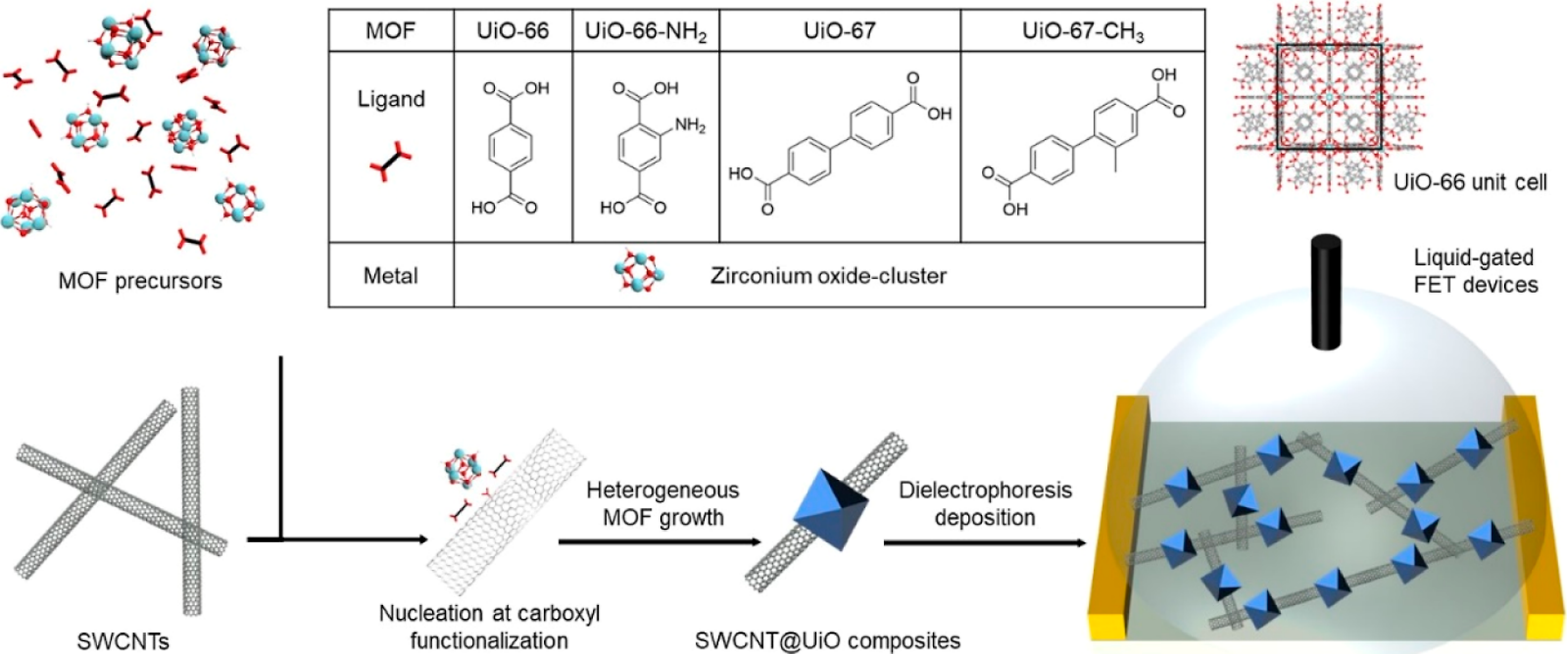
Illustration of Synthetic Strategy of SWCNT@UiO-MOF Composites and the Fabrication of Size-Based SWCNT@MOF FET Device

**Figure 1:**
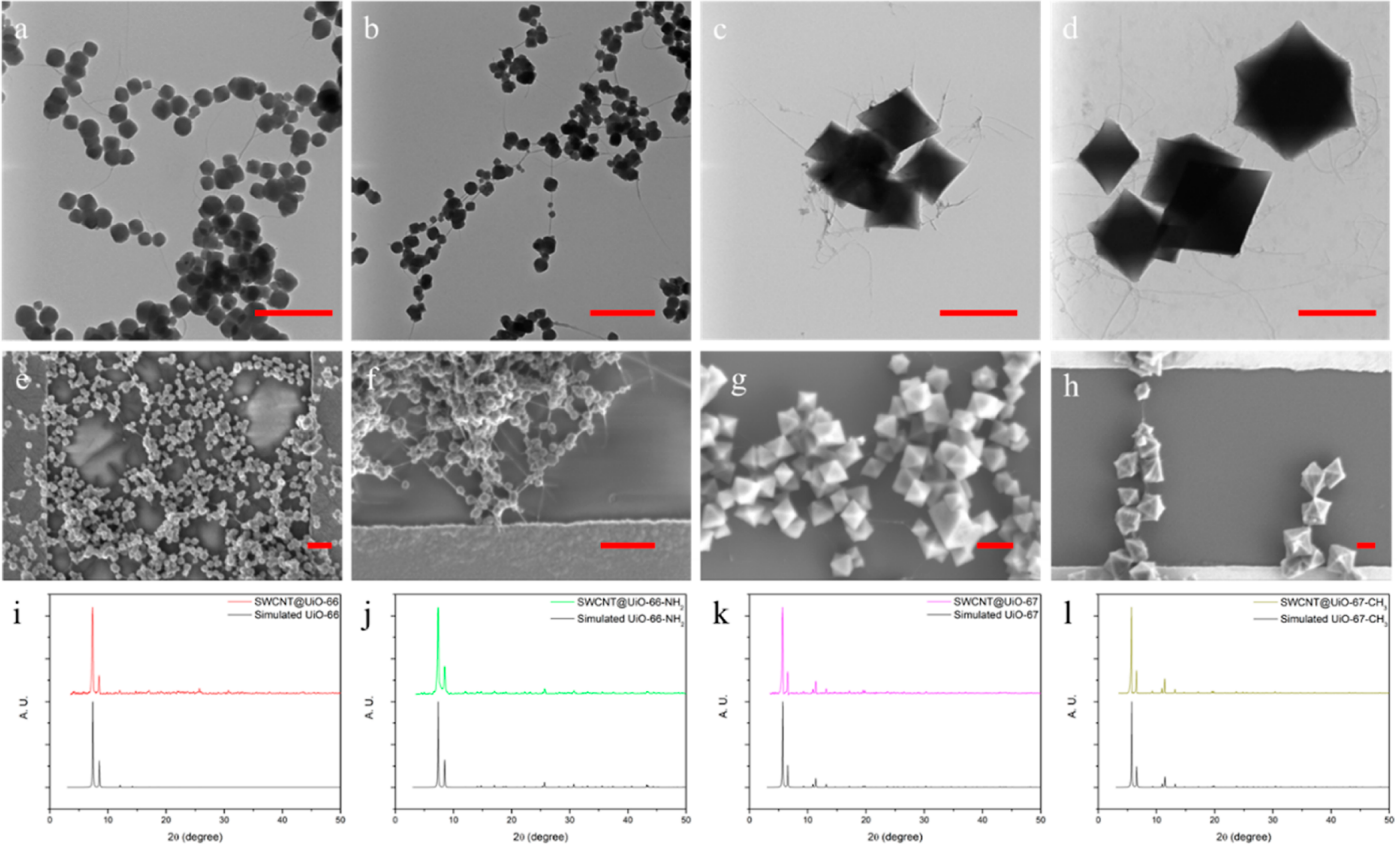
TEM images of composites (a) SWCNT@UiO-66, (b) SWCNT@UiO-66-NH_2_, (c) SWCNT@UiO-67, and (d) SWCNT@UiO-67-CH_3_; SEM images of deposited composites on interdigitated electrodes (e) SWCNT@UiO-66, (f) SWCNT@UiO-66-NH_2_, (g) SWCNT@UiO-67, and (h) SWCNT@UiO-67-CH_3_; and PXRD of composites (i) SWCNT@UiO-66, (j) SWCNT@UiO-66-NH_2_, (k) SWCNT@UiO-67, and (l) SWCNT@UiO-67-CH_3_. Scale bars are 500 nm.

Four different UiO-MOFs: UiO-66, UiO-66-NH_2_, UiO-67, and UiO-67-CH_3_, were hybridized with SWCNT. Composites were prepared by growing the MOF on the surface of SWCNT. As shown in Scheme 1, zirconium oxide-cluster and ligands tend to be adsorbed around the carboxylic functionalities on SWCNT, promoting heterogeneous MOF growth.^25^ As the TEM images indicated in Figure 1a–d, the synthesized composites shared a similar “beads-on-a-string” morphology. Composites were deposited onto prefabricated interdigitated electrodes (Figure S1) via dielectrophoresis (DEP) to fabricate FET sensors. Scanning electron microscopy (SEM) imaging (Figure 1e–h) showed that the composites formed networks to bridge the electrodes. In Figure 1i–l, the powder X-ray diffraction (XRD) patterns of the composites matched with the simulated MOF patterns, which indicated the MOF crystal structure in the composites. Comparison with pure MOFs (Figure S2) revealed that the morphology and XRD patterns of SWCNT@MOF composites remained largely unchanged, suggesting that the fundamental chemistry of the MOF component was preserved during the one-pot synthesis with SWCNTs. N_2_ sorption isotherms at 77 K were collected for the SWCNT@UiO-MOF samples (Figure S3a–d). From these data, we calculated Brunauer–Emmett–Teller (BET) surface area (SA) values of 1532 m^2^/g for SWCNT@UiO-66, 1330 m^2^/g for SWCNT@UiO-66-NH_2_, 2603 m^2^/g for SWCNT@UiO-67, and 2284 m^2^/g for SWCNT@UiO-67-CH_3_. Compared to SWCNT@UiO-66, SWCNT@UiO-66-NH_2_ possesses a smaller BET SA, likely due to the addition of the –NH_2_ functional group. Similarly, a lower BET SA is also observed for SWCNT@UiO-67-CH_3_ compared to SWCNT@UiO-67 because of the presence of –CH_3._ The calculated BET SAs for the composite materials are uniformly higher than the MOFs alone (Figure S2) and are significantly higher than what would be expected for the pure, defect-free MOFs.^46^ We note that it is not uncommon for UiO MOFs to exhibit defects,^46^ however, and that the MOF and composite syntheses reported herein likely result in some level of defects. Similar BET SA increases were also observed in other MOFs after hybridization with SWCNTs.^17,26,47,48^ The precise mechanism behind these BET surface area increases is a complex matter beyond the scope of the present work.

Pore sizes within the UiO-MOF structures were calculated using Zeo++ software (Table 1). The methodology involved employing Voronoi tessellation, a technique implemented in Zeo++, to delineate and characterize the void spaces within the MOF framework.^49^ Voronoi tessellation partitioned the space surrounding each atom into Voronoi cells, and these cells were analyzed to identify the interconnected pore channels. Pore sizes were subsequently calculated by measuring the diameters or radii of these channels. Particularly, the largest pore diameter (or the largest included sphere) corresponds to the greatest distance assigned to the Voronoi nodes. The procedure involves scanning all Voronoi nodes within a periodic unit cell of the structure and identifying the node with the largest separation from its neighboring atom. Examination of the Voronoi network also yields insights into the dimensions of the largest spherical probe capable of freely traversing the void space freely. This analysis entails an exploration of the connectivity among Voronoi nodes. To illustrate, the determination of the diameter of the largest spherical probe capable of transiting between two nodes necessitates the identification of a path within the Voronoi network that traverses nodes and edges distinguished by maximal distances from adjacent atoms. This path encompasses the regions within the void space distinguished by their widest apertures.

**Table 1:** Pore Sizes and Pore Aperture Sizes of Involved UiO-MOFs [Calculated Using Zeo++ (Version 0.3)]

MOF	pore size/Å	pore aperture size/Å
UiO-66	8.6	3.8
UiO-66-NH_2_	7.3	3.6
UiO-67	13.0	6.7
UiO-67-CH_3_	10.5	6.3

**Figure 2:**
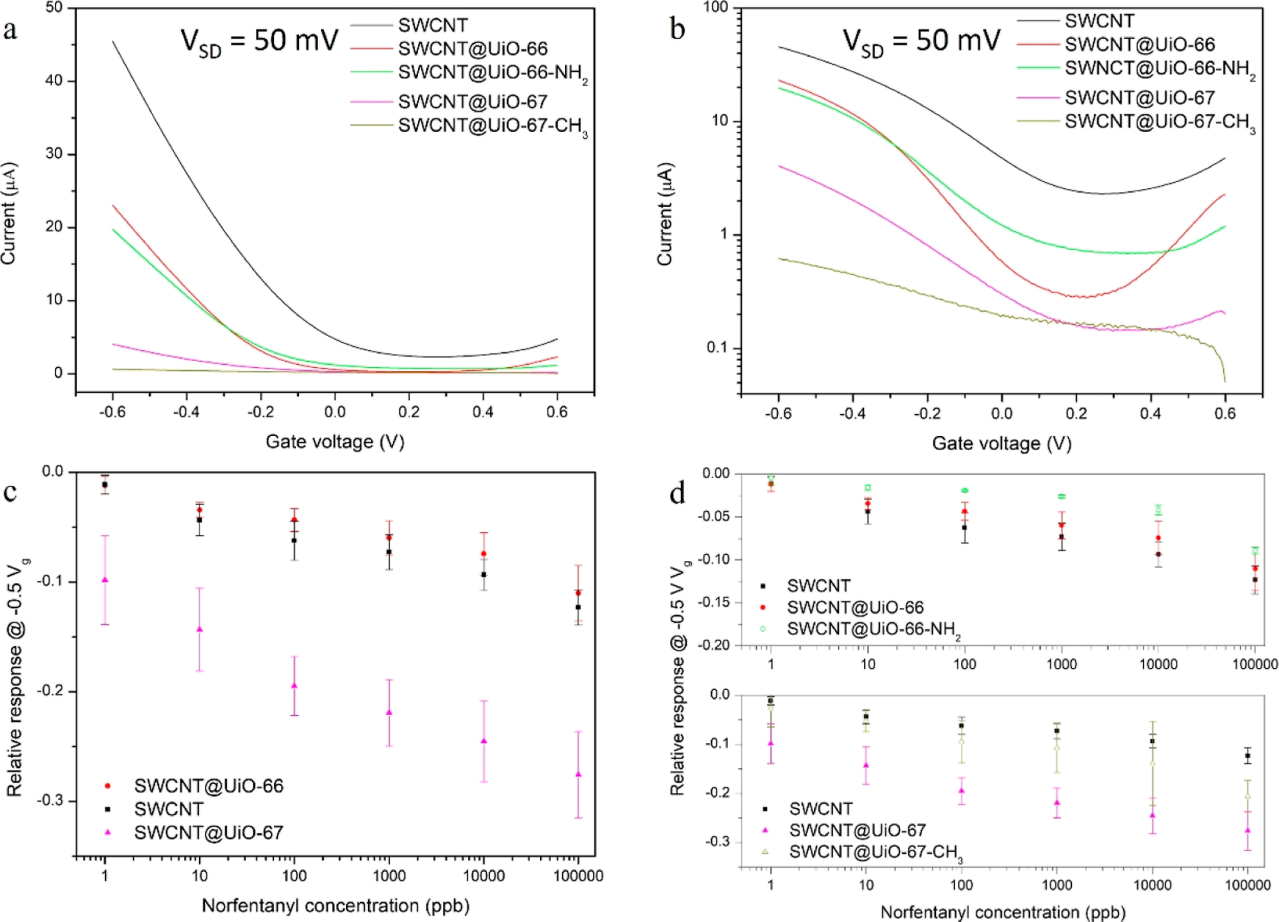
(a) Liquid-gated FET transfer characteristics (*I*–*V*_g_) of the SWCNT and composites. Source-drain voltage *V*_SD_ = 50 mV. (b) *I*–*V*_g_ curves in log scale. (c) Mean ± SD relative responses@– 0.5 V V_g_ of SWCNT (*n* = 6), SWCNT@UiO-66 (*n* = 6), and SWCNT@UiO-67 (*n* = 4). (d) Mean ± SD relative responses at −0.5 V V_g_ of SWCNT (*n* = 6), SWCNT@UiO-66 (*n* = 6), SWCNT@UiO-66-NH_2_ (*n* = 4), SWCNT@UiO-67 (*n* = 6), and SWCNT@UiO-67-CH_3_ (*n* = 6). Error bars are device-to-device variance.

Liquid-gated FET measurements were performed with composites and SWCNT devices with phosphate-buffered saline (PBS) as the gating liquid to ensure identical ionic strength. FET transfer characteristics, i.e., source-drain current versus applied gate voltage (I–V_g_), of all four composites and SWCNT devices are shown in Figure 2a. The curves of four composites all shifted toward negative voltage, suggesting the same doping effect of the UiO MOF. In Figure 2b, the transfer characteristics were plotted on a logarithmic scale. Compared to SWCNT devices, all composites showed lower off-current, which can be ascribed to the coverage of SWCNT surfaces by MOF crystals. This phenomenon can be elucidated by understanding the DEP process, which exerts forces on SWCNT strands rather than MOF particles. When the same DEP condition was applied to the composites, less material was attracted onto the electrode compared to bare SWCNTs. This resulted in a reduced availability of SWCNTs for network formation, consequently making material deposition less effective for the composites. It was noticeable that the on–off ratio of SWCNT@UiO-66 composites was slightly higher than that of bare SWCNT. This phenomenon was also observed in our previous studies with SWCNT@MOF composites, where MOF growth on carbon nanotubes suppresses the metallic aspect of the deposited nanotubes by decreasing the metallic junctions in nanotube network.^24−26^

**Figure 3:**
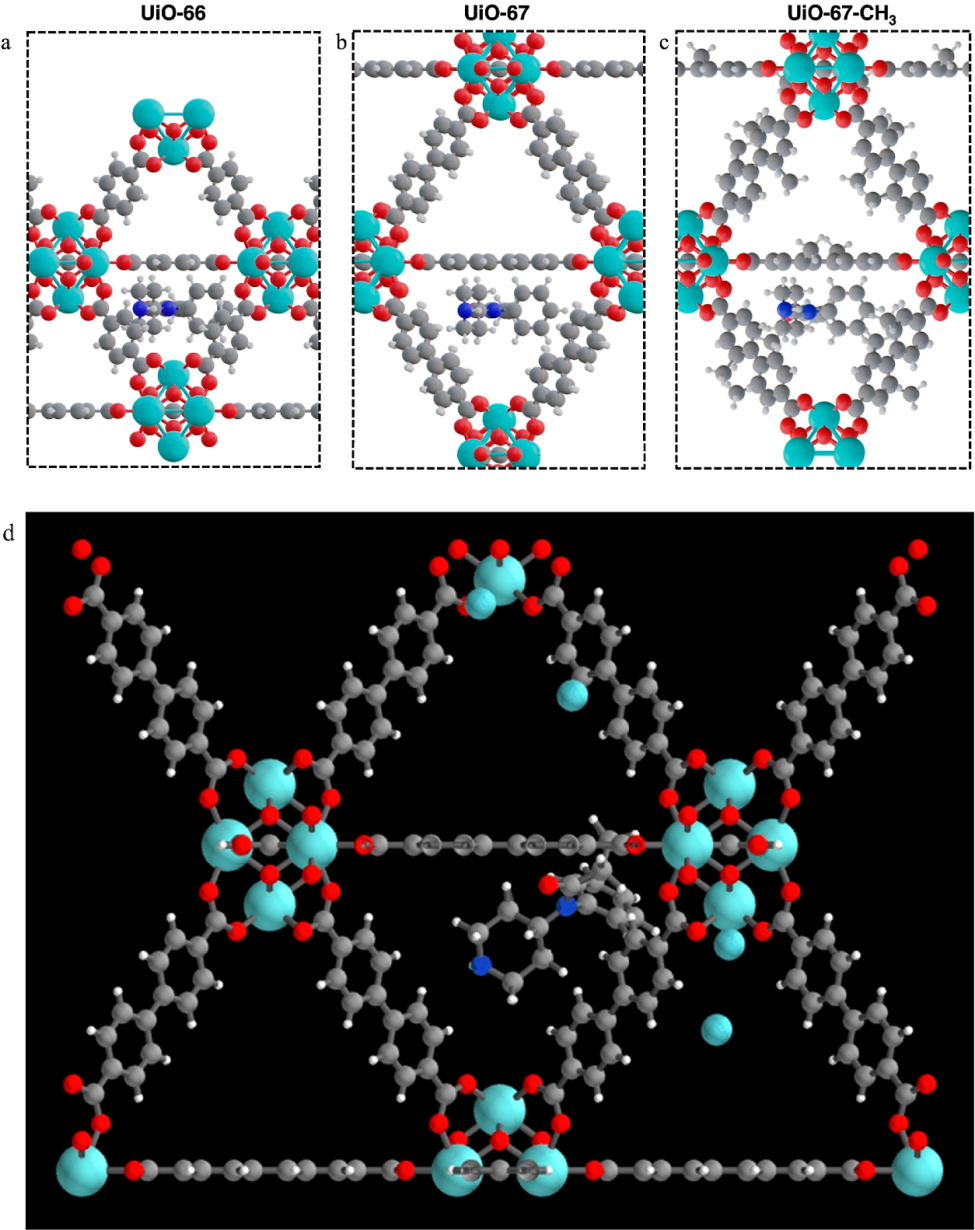
Depictions of norfentanyl molecules inside the pores of (a) UiO-66, (b) UiO-67, and (c) UiO-67-CH_3_. (d) Molecular simulation snapshot of norfentanyl in the center of the UiO-67 pore. Small turquoise spheres represent the ions in the solvent.

**Figure 4:**
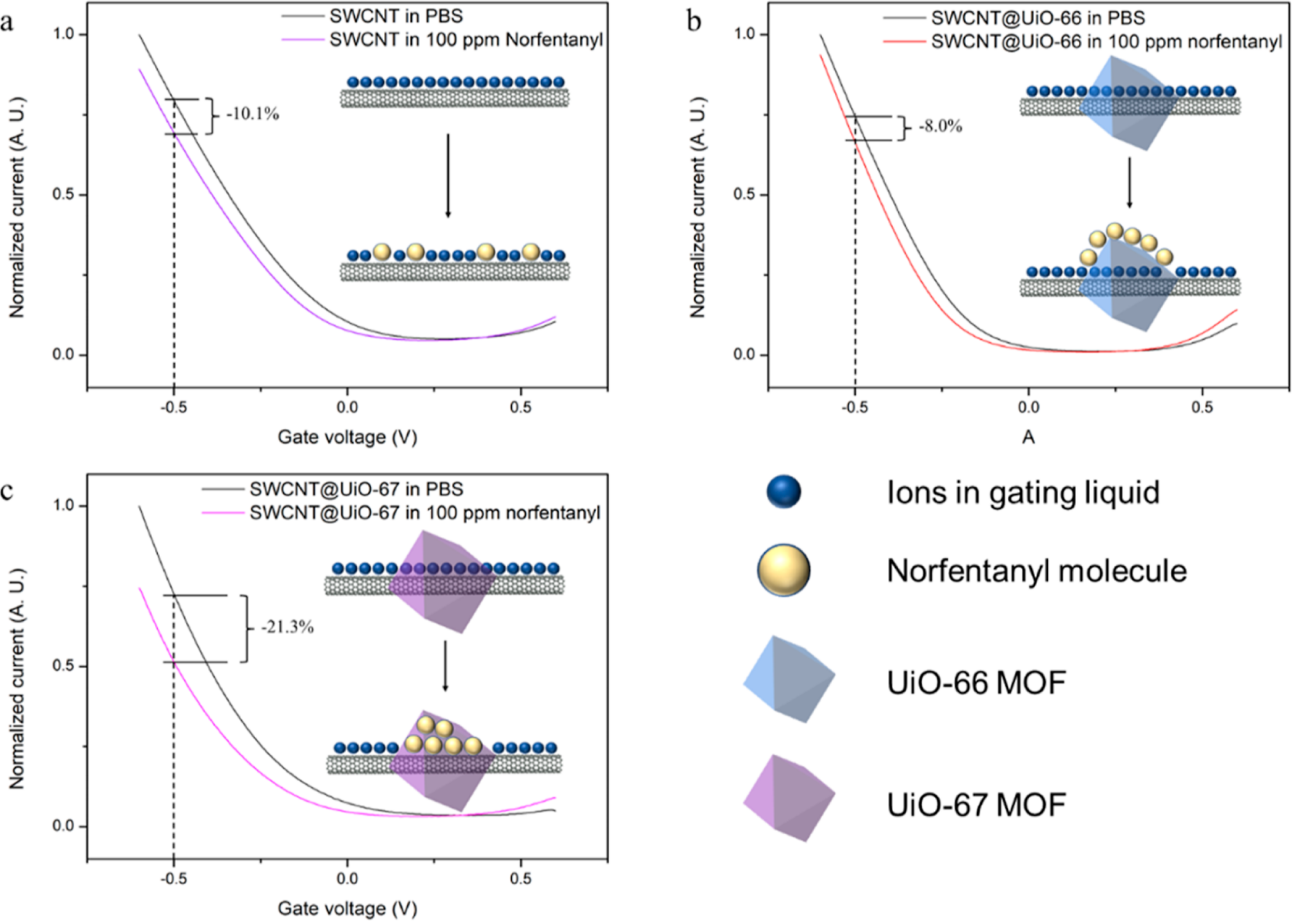
*I*–*V*_g_ curves of (a) SWCNT, (b) SWCNT@UiO-66, and (c) SWCNT@UiO-67 FET devices in PBS and 100 ppm of norfentanyl solution.

Figure 2c,d shows calibration plots depicting the responses of SWCNT devices and SWCNT@UiO-MOF composite devices when exposed to NF in PBS. The relative response was calculated by normalizing the decrease in the source-drain current at a −0.5 V gate voltage (Figure S4a). Compared to bare SWCNT devices, SWCNT@UiO-67 devices exhibited enhanced responses, while SWCNT@UiO-66 devices demonstrated decreased responses (Figure 2c). The varied responses of SWCNT@UiO-66 and SWCNT@UiO-67 devices were attributed to the pore size difference between UiO-66 and UiO-67. NF molecules were estimated to be 9.6 Å in size (Figure S5). As shown in Table 1, the pore size of UiO-66 is 8.6 Å, which is too small for the NF molecule to enter (Figure 3a). However, in the case of UiO-67, NF molecules can enter the MOF pores, as depicted in Figure 3b,d. Figure 3d presents a snapshot from a molecular simulation depicting NF molecules residing in the center of the UiO-67 pores. For a dynamic view of NF molecules moving within the pores, a video is available in the Supporting Information. In a liquid-gated FET, a SWCNT relies on the gate-voltage-driven diffusion of ions to modulate its conductance. When NF molecules resided inside the channel of UiO-67, ion diffusion was partially obstructed, leading to a decrease in the gate capacitance of the FET device. Consequently, there was a significant current reduction in the p-branch of the *I*–*V*_g_ curve as depicted in Figure 4c. For bare SWCNT devices, decrease in the current resulted from nonpreferential adsorption of NF on SWCNT surfaces. These adsorbed molecules can also hinder ion diffusion, causing a decrease in the current. However, this effect was less prominent compared with the blockage inside MOF channels. In the case of the SWCNT@UiO-66 devices, NF molecules cannot enter UiO-66. Only the exposed SWCNTs in the SWCNT@UiO-66 devices were subjected to NF molecules. As a result, the influence of NF on SWCNT@UiO-66 devices was less pronounced compared to bare SWCNT devices (Figure 4b). While it is true that adsorbed NF molecules may induce changes in SWCNT conductance through an electrostatic effect, this effect was universal throughout all devices. Therefore, this factor was not discussed when comparing the differences in responses.

UiO-66 and UiO-67 can be functionalized on the ligand. With one substitution on the benzene ring, the pore size can be slightly decreased (Table 1). UiO-66-NH_2_ and UiO-67-CH_3_ have an amine and a methyl group on their ligands, respectively. Their composites with the SWCNT were also tested with NF (Figure 2d). SWCNT@UiO-66-NH_2_ composite devices showed slightly lower responses compared to those of SWCNT@UiO-66 devices. SWCNT@UiO-66 devices have already exhibited reduced responses compared to bare SWCNT devices, primarily due to the prevention of interaction between NF and SWCNT. Further decreasing the MOF pore size should not significantly alter this outcome. The variation in responses was attributed to the slight difference in the MOF coverage between SWCNT@UiO-66 and SWCNT@UiO-66-NH_2_ composites. SWCNT@UiO-67-CH_3_ composite devices showed increased responses compared to SWCNT devices, but the enhancement of responses was smaller than that of the SWCNT@UiO-67 devices. We propose that the decreased pore size in UiO-67-CH_3_ increases the steric hindrance felt by NF molecules, which slows their diffusion into the MOF (Figure 3c), leaving more channels open for ions to reach the SWCNTs after the devices were incubated for the same time.

The stability of the SWCNT@UiO-67 composites was assessed by storing and aging them in ethanol. XRD and SEM images were obtained at two time points: 65 and 226 days. For the material aged for 65 days, no significant decrease in crystallinity was observed in the XRD pattern (Figure S6), and SEM images showed no noticeable changes in morphology (Figure S7a). However, in the 226 day sample, a substantial reduction in the XRD peak intensity was evident (Figure S6), and SEM images revealed visible degradation, including surface holes on UiO-67 particles (Figure S7b), indicative of structural damage. FET devices were fabricated with 226 day aged composites, and their responses were tested against NF (Figure S8). Despite significant MOF structure degradation, the FET devices still exhibited enhanced responses toward NF. This continued response can be attributed to the presence of remaining crystallized MOF channels within the composites, which NF can still occupy and obstruct ion diffusion. The reduced enhancement was attributed to reduced availability of UiO-67 channels in the degraded composites. Signal saturation was observed around 100 ppb to 1 ppm region, possibly due to the limited amount of MOF pores after degradation. An additional linear region appeared in the ppm range, but due to the increased complexity of the MOF structure after degradation, it was challenging to rationalize this observation within the scope of this study. The stability of the fabricated devices was assessed by comparing the sensor response before and after storage in a drawer under ambient conditions for 36 days. As shown in Figure S8, there is a negligible difference between the devices tested immediately after fabrication and those tested after storage, indicating the good stability of the sensor devices after fabrication.

Metabolites of other controlled substances were also tested to investigate the sensing specificity of the composites. Normorphine (NM), norhydrocodone (NH), and benzoylecgonine (BZ) are major metabolites of morphine, hydrocodone, and cocaine, respectively. These compounds were dissolved in PBS and added to the SWCNT@UiO-67 device with concentrations ranging from 1 ppb to 1 ppm (Figure S9). The sensor devices showed no response to NH and BZ, but showed similar responses toward NM because its size is similar to NF. The responses to other metabolites with similar size to NF proved the sensing mechanism was based on the size of the molecule.

When compared with other sensing methods, the limit of detection (LOD) from direct measurement using SWCNT@MOF FET sensors is not the lowest (Table S1). However, SWCNT@MOF FET sensors offer a versatile platform for detecting analytes that are challenging for conventional chemical sensing methods by leveraging size-based detection. By incorporating such sensors into arrays and applying machine learning methods, discernment between chemically similar analytes can be achieved.^50−52^ Prior work has shown using such methods can lead to improvements in sensitivity and selectivity,^53−55^ even for materials that were once considered nonspecific.^56^ Furthermore, discrimination of analytes in complex chemical environments has been demonstrated.^57−59^ Our exploration of these SWCNT@MOF FET materials has only just begun, leaving many aspects to investigate, such as the chemical interaction between functional groups inside the MOF channel and analyte molecules, sensor fabrication methods, and optimization of pore size for specific analytes.

## Conclusions

The heterogeneous growth of four different UiO-MOFs, namely, UiO-66, UiO-66-NH_2_, UiO-67, and UiO-67-CH_3_, on SWCNTs was demonstrated. The resulting SWCNT@UiO-MOF materials effectively combined porosity and electrical conductivity. These materials were subsequently fabricated into liquid-gated FET devices, marking the first instance of NF detection without the need for the sensor functionalization with biorecognition elements, such as aptamer or antibody. SWCNT@UiO-67 and SWCNT@UiO-67-CH_3_ demonstrated concentration-based responses to NF in PBS solution, validating the size-based sensing mechanism proposed for the SWCNT@MOF FET sensors. Due to the matched size of the MOF pores and NF molecules, SWCNT@UiO-67 exhibited the best response among the tested SWCNT@UiO-MOF devices. A comparison between SWCNT@UiO-67 and SWCNT@UiO-67-CH_3_ devices indicated that the sensor response was also related to the diffusion of NF into the MOF channel because of the incubation-based testing method. Three metabolites of different drugs were also tested, and the sensor device effectively screened out interference molecules with sizes larger than the MOF pores. However, since the sensor responded solely based on the size of the analyte, achieving specificity toward a single analyte cannot be accomplished with one type of SWCNT@MOF sensor alone. More SWCNT@MOF composites need to be synthesized to construct a sensor array with different pore sizes and channel chemistries to improve the selectivity of the SWCNT@MOF FET sensing platform. With the assistance of machine learning-driven discrimination, such sensor arrays hold the potential to generate unique signals for different analytes, enabling molecule identification in future work.

## Experimental Section

### Preparation of SWCNT Stock Suspension

SWCNTs (P3-SWNT, Carbon Solutions, Inc.) were prepared into 0.5 mg/mL solution of DMF. The solution was sonicated for 1 h to suspend SWCNTs. For each synthesis, SWCNT suspension was sonicated for 15 min before use.

### Preparation of Zr Oxide-Cluster Solution

Seven mL of DMF was mixed with 4 mL of acetic acid, followed by 71 μL of 70 wt % zirconium propoxide solution in 1-propanol. The mixture was heated in an oven at 130 °C for 2 h until the solution appeared pale yellow. The resulting mixture was cooled to room temperature.

### Synthesis of SWCNT@UiO-MOF Composites

Syntheses of UiO-MOFs were adapted from published methods.^60,61^ Briefly, the SWCNT suspension was mixed with the MOF precursor solution. An oil bath was used for the heated reaction. Detailed amount and method for each composite is included in the Supporting Information.

### Fabrication of FET Devices

Composites and SWCNTs were deposited on prefabricated interdigitated electrodes by using dielectrophoresis (DEP). The prefabricated interdigitated electrode area is 300 × 200 μm. Channels between the electrodes are 6 μm (Figure S1). A Keithley 3390 Arbitrary Waveform Generator was used to generate a sine wave (10 V_pp_, 10 MHz). Three μL of samples suspension (0.5 mg/mL for composites, 0.1 mg/mL for SWCNT) was drop-cast on the device, and the sine wave was applied to the electrodes for 5 min. The same procedure was performed 2–3 times until conductive devices were achieved. The deposited devices were washed with water and then annealed at 200 °C for 1 h to remove the solvent residue.

### FET Sensing of Norfentanyl

Norfentanyl solutions with concentrations from 1 ppb to 100 ppm were prepared by dissolving NF in PBS solution. FET measurements were performed using 300 μL of PBS or NF solution as liquid gating media. An Ag/AgCl reference electrode was in contact with the gating liquid. 50 mV bias voltage was applied to source-drain channel, while gate voltage swept from 0.6 to −0.6 V. All devices were stabilized with PBS solution until their FET curves remained the same after changing the gating PBS solution. After each measurement, devices were rinsed with DI water and blown dry with N_2_ before the next gating liquid was added. Devices were incubated in gating liquid for 10 min before measurement. FET measurements were made with Keithley 2400 source meter units.

## Supporting Information Available

Experimental methods, nitrogen adsorption isotherms, FET curves, and additional drug metabolites calibration plots (PDF)
Dynamic view of NF molecules moving within the pores (MP4)
