# Supplementary material for: Size-Based Norfentanyl Detection with SWCNT@UiO-MOF Composites
Source: ACS Appl Mater Interfaces. 2023 Dec 26;16(1):1361–9. doi: 10.1021/acsami.3c17503 (PMC10788826; doi:10.1021/acsami.3c17503)
Supplement: Supplementary file 1 — am3c17503_si_001.pdf [file am3c17503_si_001.pdf]

Supporting Information

Size-based norfentanyl detection with SWCNT@UiO-MOF composites

Zidao Zeng[a], Meiirbek Islamov[b], Yiwen He[a,b], Brian A. Day[b], Nathaniel L Rosi[a,b], Christopher E. Wilmer[b,c,e], Alexander Star[a,d,e]*

[a] Department of Chemistry, University of Pittsburgh, Pittsburgh, Pennsylvania 15260, United States

[b] Department of Chemical & Petroleum Engineering, University of Pittsburgh, Pittsburgh, Pennsylvania 15260, United States

[c] Department of Electrical & Computer Engineering, University of Pittsburgh, Pittsburgh, Pennsylvania 15260, United States

[d] Department of Bioengineering, University of Pittsburgh, Pittsburgh, Pennsylvania 15260, United States

[e] Clinical and Translational Science Institute, University of Pittsburgh, Pittsburgh, Pennsylvania 15260, United States

Correspondence to: astar@pitt.edu

Table of content

Additional experimental details……………………………………………...……………… S-3

Figure S1. SEM image of the interdigitated electrode device ...……………………………...S-5

Figure S2. Characterization of UiO-MOFs (TEM, XRD, N_2 isotherm) ...……………….……...S-6

Figure S3. N_2 sorption isotherms of SWCNT@UiO-MOF composites.......................……......S-7

Figure S4. Typical I-V_g curves and calibration plot of SWCNT@UiO-67 device......................S-8

Figure S5. Optimized model of involved drug metabolites and their sizes..................................S-9

Figure S6. PXRD pattern of new and aged SWCNT@UiO-67 composites..............................S-10

Figure S7. SEM images of aged SWCNT@UiO-67 composites..S-11

Figure S8. New and aged SWCNT@UiO-67 FET sensor responses toward norfentanyl..........S-12

Figure S9. Responses of SWCNT@UiO-67 devices toward other drug metabolites S-13

Table S1. Comparison of published norfentanyl detection methods and this work……………S-14

References..…………………………………………………………………………………S-15

Additional Experimental Details

Chemicals and Materials

Zirconium (IV) propoxide solution (70 wt. % in 1-propanol) (Sigma-Aldrich), Zirconium (IV) chloride anhydrate (Sigma-Aldrich), terephthalic acid (Sigma-Aldrich), 2-aminoterephthalic acid (Alfa Aesar), biphenyl-4,4'-dicarboxylic acid (Sigma-Aldrich), acetic acid (Sigma-Aldrich), N,N-dimethylformamide (Sigma-Aldrich), ethylene glycol dimethyl ether (Sigma-Aldrich), methyl 4-iodo-3-methylbenzoate (Sigma-Aldrich), 4-methoxyl carbonylphenylboronic acid (Sigma-Aldrich), potassium carbonate (Sigma-Aldrich), tetrakis (triphenylphosphine) palladium (Sigma-Aldrich), SWCNT (P3-SWNT, Carbon Solutions, Inc) were purchased and used without further purification.

Characterization

Transmission Electron Microscopy (TEM). FEI Morgagni was used for taking TEM images.

Scanning Electron Microscopy (SEM). SEM images were taken on a Zeiss Sigma 500 VP analytical FE-SWM equipment.

Powder X-ray Diffraction (PXRD). PXRD was conducted with Bruker D8 XRD system equipped with LynxEye detector. Samples were prepared by drop-casting on glass slides. 2θ angles between 3.5° and 50° were measured at 0.02° interval with a rate of 0.3 seconds/point. The X-ray source was Cu Kα held at 40 kV and 40 μA.

Gas adsorption isotherms were collected on a Micromeritics 3-flex gas adsorption analyzer. Approximately 20 mg of each sample was added into a pre-weighed sample analysis tube that had been evacuated and backfilled with N_2 before massing. The samples were degassed at 423 K under vacuum for 24 hours until the pressure change rate was no more than 3.5 mTorr/min. A liquid N_2 bath was used for the N_2 adsorption experiments at 77 K. UHP grade N_2 adsorbate (99.999 %) was used in this study.

Experimental details

Synthesis of 2-methyl-biphenyl-4,4'-dicarboxylic acid (MeBPDC). MeBPDC was synthesized according to literature conditions.[1]

Synthesis of SWCNT@UiO-66. To a 20 mL scintillation vial, 12.18 mL DMF, 3.73 mg terephthalic acid, 0.40 mL acetic acid and 0.32 mL SWCNT suspension (0.5 mg/mL) were added. The mixture was sonicated for 1 minute before 1.10 mL Zr oxide-cluster solution was added. The mixture was kept at room temperature under stirring for 20 hours before the composites were recovered using centrifugation. Composites were washed with DMF and ethanol three times and were stored in ethanol.

Synthesis of SWCNT@UiO-66-NH_2. To a 20 mL scintillation vial, 12.18 mL DMF, 4.07 mg 2-aminoterephthalic acid, 0.40 mL acetic acid and 0.32 mL SWCNT suspension (0.5 mg/mL) were added. The mixture was sonicated for 1 minute before 1.10 mL Zr oxide-cluster solution was added. The mixture was kept at room temperature under stirring for 20 hours before the composites were recovered using centrifugation. Composites were washed with DMF and ethanol three times and were stored in ethanol.

Synthesis of SWCNT@UiO-67. To a 20 mL scintillation vial, 12.75 mL DMF and 24.23 mg biphenyl-4,4'-dicarboxylic acid (BPDC) was added. The solution was heated at 150°C in an oil bath to dissolve BPDC. After cooling the solution to room temperature, 23.30 mg $ZrCl_4$, 1.75 mL acetic acid and 0.50 mL SWCNT suspension (0.5 mg/mL) was added. The solution was sonicated for 1 minute and then kept in a 120°C oil bath for 5 hours under stirring. The yield composites were recovered using centrifugation and were washed with DMF and ethanol three times. Composites were stored in ethanol.

Synthesis of SWCNT@UiO-67-CH_3. To a 20 mL scintillation vial, 9.74 mL DMF, 0.50 mL acetic acid and 9.32 mg $ZrCl_4$ were added. The solution was sonicated for 1 minute and then kept in a 100°C oil bath for 10 minutes. The transparent solution was then cooled to room temperature and 10.24 mg MeBPDC and 0.26 mL SWCNT suspension (0.5 mg/mL) were added. The mixture was kept in a 100°C oil bath for 20 hours under stirring. The composites were recovered using centrifugation and were washed with DMF and ethanol three times. Composites were stored in ethanol.

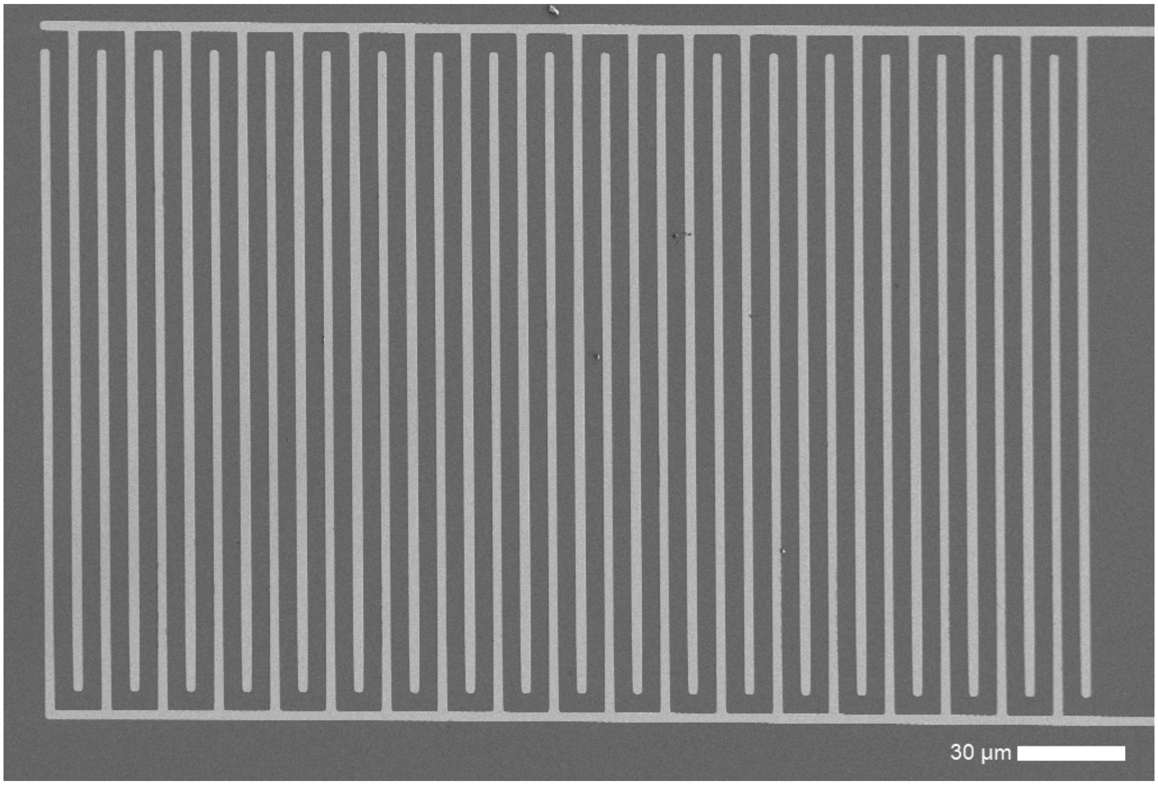

Figure S1. SEM image of the interdigitated electrode device. The dimension of the electrode area is 300 µm x 200 µm. Distance between each electrode finger is 6 µm.

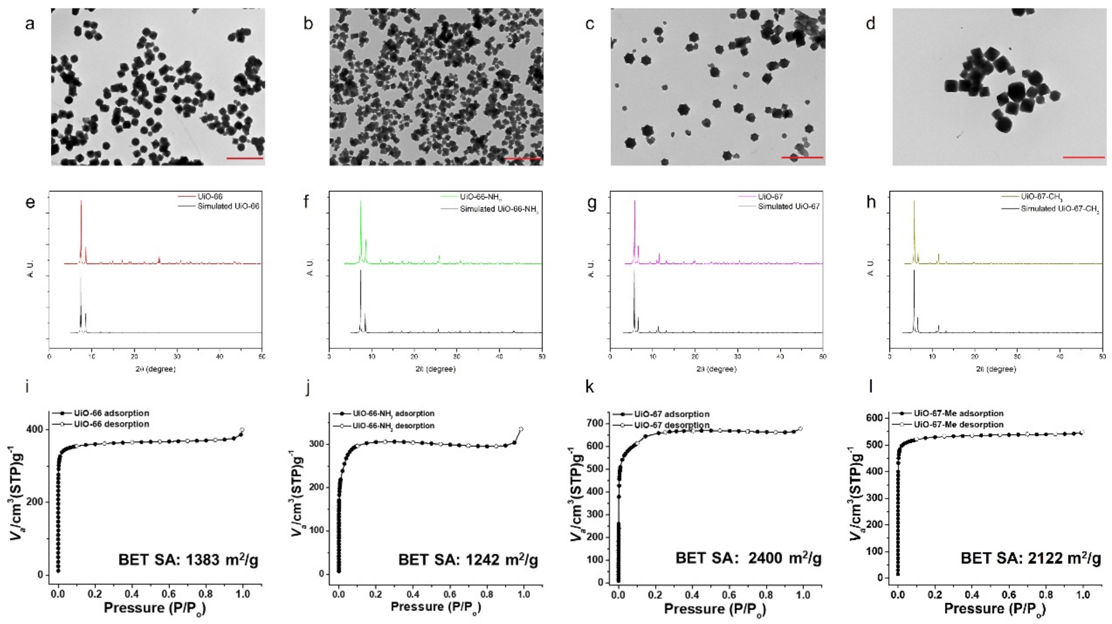

Figure S2. Characterization of UiO-MOFs. TEM images of (a) UiO-66, (b) UiO-66-NH_2, (c) UiO-67 and (d) UiO-67-CH_3, scale bars are 1 μm; PXRD of MOF (e) UiO-66, (f) UiO-66-NH_2, (g) UiO-67 and (h) UiO-67-CH_3; N_2 sorption isotherms at 77 K of (i) UiO-66, (j) UiO-66-NH_2, (k) UiO-67 and (l) UiO-67-CH_3.

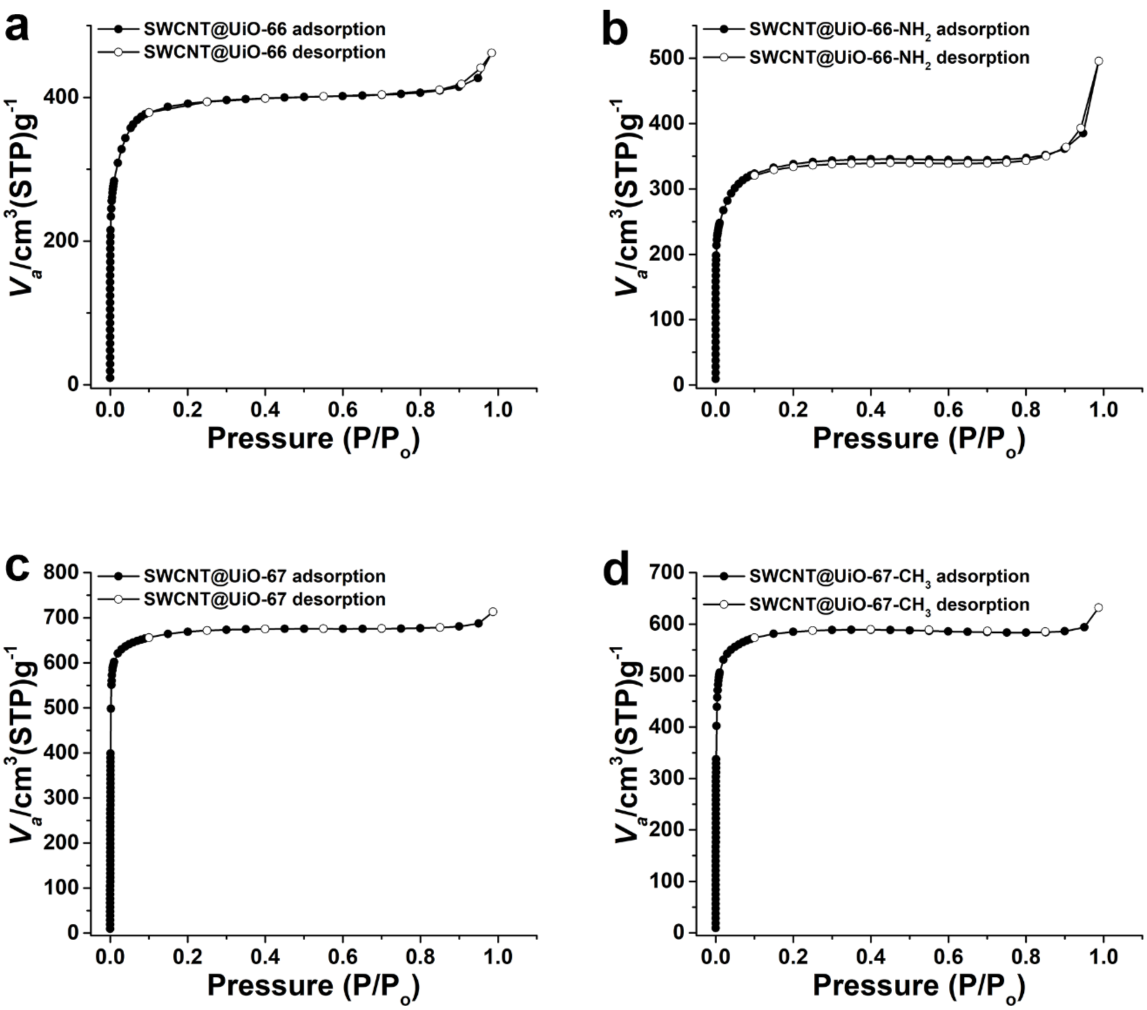

Figure S3. N_2 sorption isotherms at 77 K of SWCNT@UiO-66 (a), SWCNT@UiO-66-NH_2 (b), SWCNT@UiO-67 (c) and SWCNT@UiO-67-CH_3 (d).

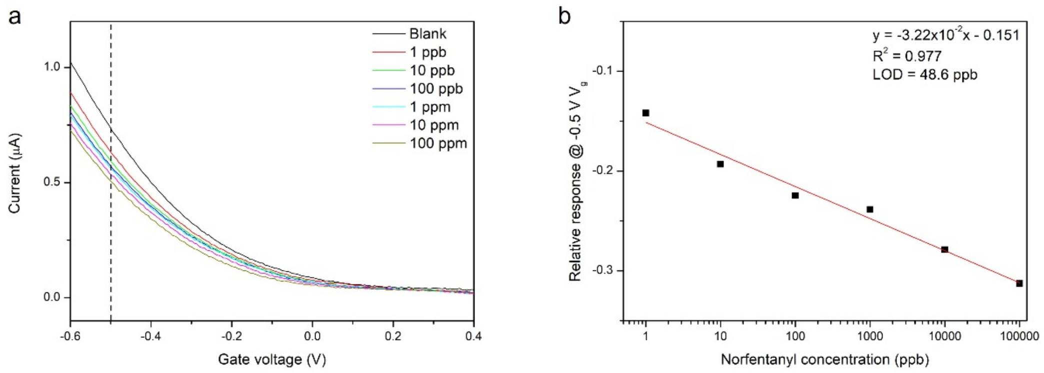

Figure S4. (a) Typical I-V_g curves of SWCNT@UiO-67 device when exposed to different concentrations of norfentanyl in PBS. Relative response is calculated by normalizing the change of current at V_g = –0.5 V to the blank curve. (b) Linear fitting for the relative response at V_g= –0.5 V. Limit of detection (LOD) was determined as LOD = 3.3σ/m, where σ is the standard deviation of y-intercept and m is the calibration sensitivity (i.e., slope of the calibration plot).

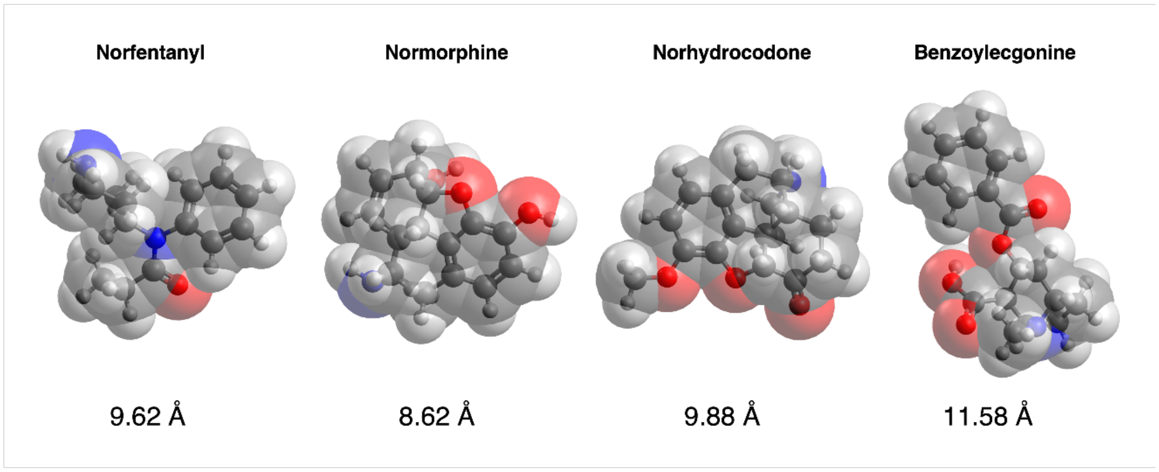

Figure S5. Optimized molecular structures of drug metabolites (NF, NM, NH, BZ) and their corresponding molecular dimensions. Molecular size is determined as the distance between the two farthest atoms within each molecule.

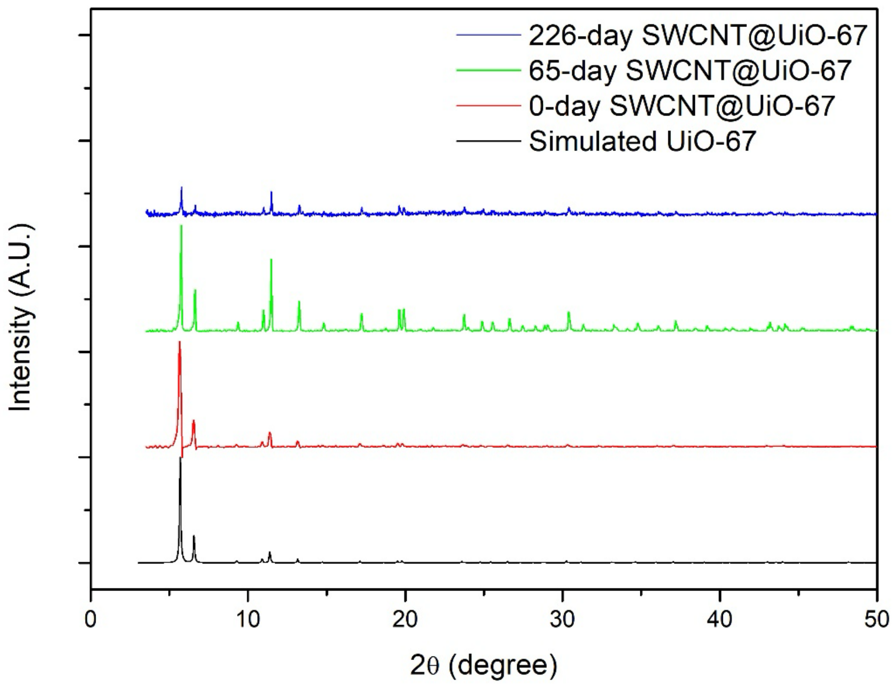

Figure S6. PXRD pattern of new and aged SWCNT@UiO-67 composites.

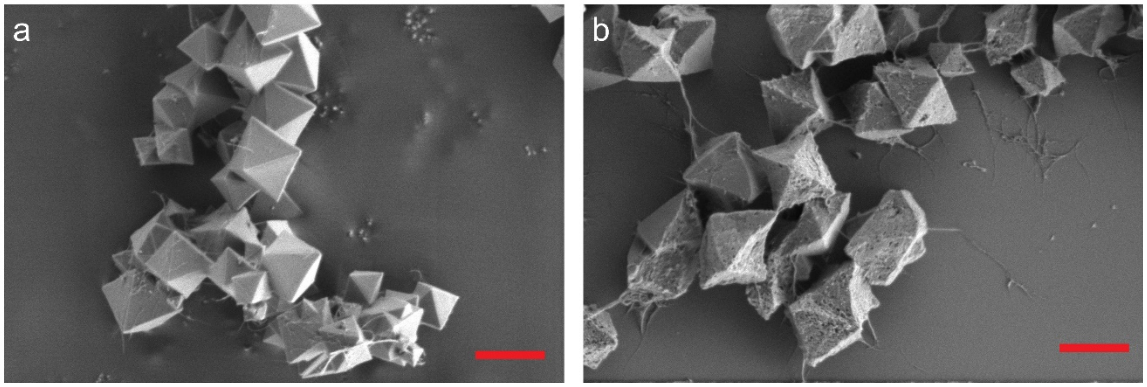

Figure S7. SEM images of aged SWCNT@UiO-67 composites. (a) 65-day composites. (b) 226-day composites. Scale bars are 1 μm.

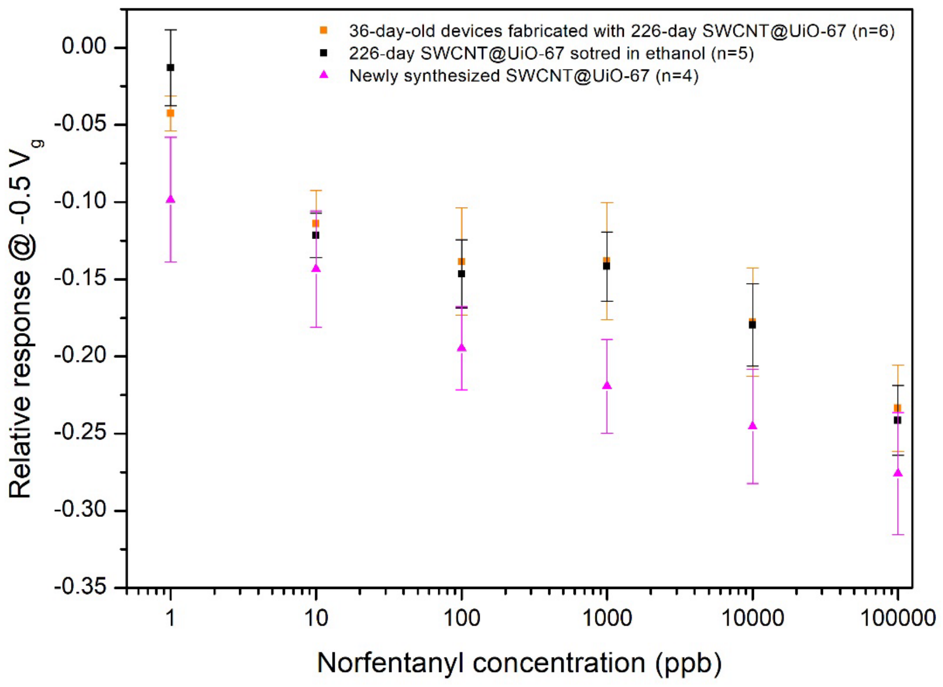

Figure S8. New and aged SWCNT@UiO-67 FET sensor responses toward norfentanyl. Error bars are device-to-device variance. Number of devices (n) is presented for each device type.

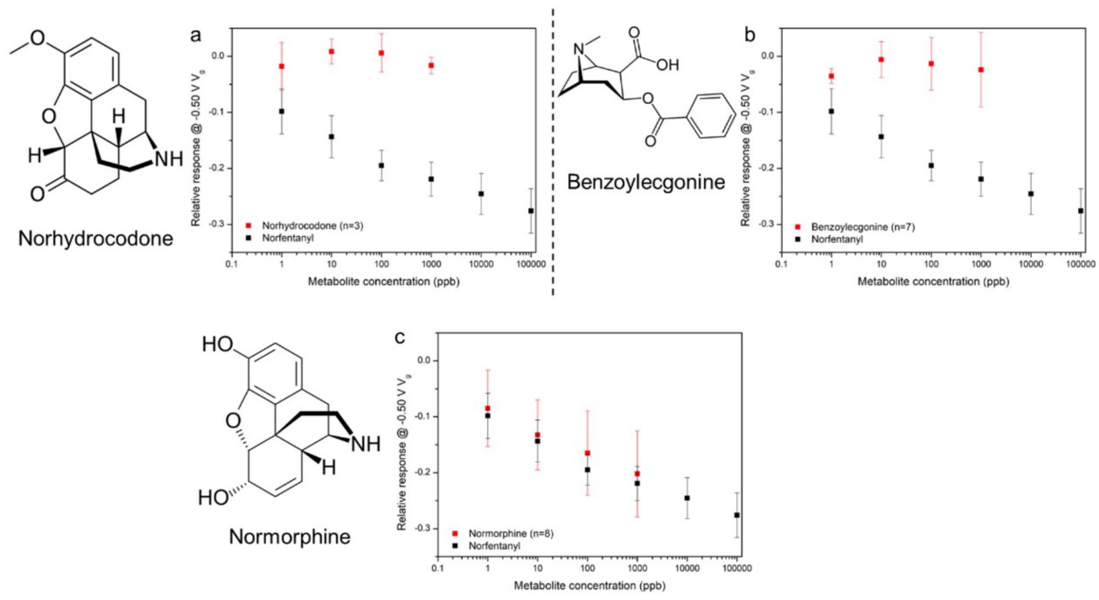

Figure S9. Responses of SWCNT@UiO-67 composites toward other drug metabolites: (a) norhydrocodone, (b) benzoylecgonine, and (c) normorphine.

Table S1. Comparison of published norfentanyl detection methods and this work.

Sensing method	Limit of detection(LOD)	Sample source	reference
lateral flow immunoassay (LFIA)	8 ng/mL	urine	2
LFIA	100 ng/ml	saliva	2
G-FET	42 pg/mL	wastewater diluted 20× in PBS	3
sc-SWCNT-FET	2.0 fg/mL<br>3.7 fg/mL	synthetic urine diluted 10x, 100x, and 1000x in PBS	4
SWCNT@MOF-FET	48 ng/mL	PBS	this work

References

(1) Boone, P., He, Y., Lieber, A. R., Steckel, J. A., Rosi, N. L., Hornbostel, K. M., Wilmer, C. E.. Designing Optimal Core-Shell MOFs for Direct Air Capture. *Nanoscale*, **2022,** *14*, 16085-16096, DOI: 10.1039/d2nr03177a.

(2) Angelini, D. J.; Biggs, T. D.; Maughan, M. N.; Feasel, M. G.; Sisco, E.; Sekowski, J. W., Evaluation of a lateral flow immunoassay for the detection of the synthetic opioid fentanyl. *Forensic Sci Int* **2019,** *300*, 75-81.

(3) Kumar, N.; Rana, M.; Geiwitz, M.; Khan, N. I.; Catalano, M.; Ortiz-Marquez, J. C.; Kitadai, H.; Weber, A.; Dweik, B.; Ling, X.; van Opijnen, T.; Argun, A. A.; Burch, K. S., Rapid, Multianalyte Detection of Opioid Metabolites in Wastewater. *ACS Nano* **2022,** *16* (3), 3704-3714.

(4) Shao, W.; Zeng, Z.; Star, A., An Ultrasensitive Norfentanyl Sensor Based on a Carbon Nanotube-Based Field-Effect Transistor for the Detection of Fentanyl Exposure. *ACS Appl Mater Interfaces* **2023,** *15* (31), 37784-37793
